# Supplementary material for: Expression Profile and Prognostic Value of Wnt Signaling Pathway Molecules in Colorectal Cancer
Source: Biomedicines. 2021 Sep 27;9(10):1331. doi: 10.3390/biomedicines9101331 (PMC8533439; doi:10.3390/biomedicines9101331)
Supplement: Supplementary file 1 [file biomedicines-09-01331-s001.zip › biomedicines-1387369-supplementary.pdf]

Expression Profile and Prognostic Value of Wnt Signaling Pathway Molecules in Colorectal Cancer

Yung-Fu Wu [1,†], Chih-Yang Wang [2,3,†], Wan-Chun Tang [2], Yu-Cheng Lee [4], Hoang Dang Khoa Ta [5], Li-Chia Lin [3], Syu-Ruei Pan [3], Yi-Chun Ni [3], Gangga Anuraga [2,5] and Kuen-Haur Lee [2,3,7,*]

[1] Department of Medical Research, Tri-Service General Hospital, School of Medicine, National Defense Medical Center, Taipei 11490, Taiwan; qrince@yahoo.com.tw
[2] PhD Program for Cancer Molecular Biology and Drug Discovery, College of Medical Science and Technology, Taipei Medical University, Taipei 11031, Taiwan; chihyang@tmu.edu.tw (C.-Y.W.); yeas0310@hotmail.com (W.-C.T.); g.anuraga@unipasby.ac.id (G.A.)
[3] Graduate Institute of Cancer Biology and Drug Discovery, College of Medical Science and Technology, Taipei Medical University, Taipei 11031, Taiwan; m654108001@tmu.edu.tw (L.-C.L); panray8802069487@gmail.com (S.-R.P.); ckni1012@gmail.com (Y.-C.N.); khlee@tmu.edu.tw (K.-H.L.)
[4] Graduate Institute of Medical Sciences, College of Medicine, Taipei Medical University, Taipei 11031, Taiwan; yclee0212@tmu.edu.tw
[5] PhD Program for Cancer Molecular Biology and Drug Discovery, College of Medical Science and Technology, Taipei Medical University and Academia Sinica, Taipei 11031, Taiwan; d621109004@tmu.edu.tw
[6] Department of Statistics, Faculty of Science and Technology, Universitas PGRI AdiBuana, Surabaya, East Java 60234, Indonesia
[7] Cancer Center, Wan Fang Hospital, Taipei Medical University, Taipei 11031, Taiwan
† These authors contributed equally to this work.
* Correspondence: khlee@tmu.edu.tw

Supplemental Material includes three Supplemental Tables and two Supplemental Figure

Supplemental Tables

Table S1. Sequences of small interfering (si)RNAs and primers.
Table S2. Top 10 up-regulated genes in CRC (GSE21815).
Table S3. Univariate and multivariate Cox regression analysis of the 10 CRC-associated genes.

Supplemental Figure

Figure S1. The expression status of top 10 upregulated CRC-associated genes in the total of 716 samples from six studies.
Figure S2. The prognostic value of mRNA expression level of CRC-associated genes in patients with CRC. The relationship between KRT80 **(A)**, FABP6 **(C)**, FOXQ1 **(B)**, CEMIP **(C)**, and GAS2 **(D)** expression and survival status in 466 TCGA-CRC patients.
Figure S3. The correlation between CTNNB1 and 3 CRC-associated genes in CRC. **(A)-(C)** There was no correlation between CTNNB1 and FABP6, ETV4, or TESC in 275 CRC patients from the TCGA database.

Supplementary Table S1. Sequences of small interfering (si)RNAs and primers

**Sequences of siRNAs**			
**Gene name**	**Sense strand (5′-3′)**	**Antisense strand (5′-3′)**	
CTNNB1	CUAUCUGUCUGCUCU AGUATT	UACUAGAGCAGACAG AUAGTT	
NKD2	GCACUCCAGUGUGAU GUCUTT	AGACAUCACACUGGA GUGCTT	
FOXQ1	CCAUCAAACGUGCCU UAAATT	UUUAAGGCACGUUUG AUGGTT	
CEMIP	GGACGGAGUGGUUCG AUCAUGAUAATT	UUAUCAUGAUCGAAC CACUCCCGUCCTT	
**Sequence of primers**			
**Gene name**	**Forward (5′-3′)**	**Reverse (5′-3′)**	**Melting Temperature (Tm) (°C)**
CTNNB1	TGCTAAATGACGAGG ACCAG	TGAGGAGAACGCATG ATAGC	54
NKD2	CCTTAGACCCCACTAC CCGA	TTTTTCGCCCAGTGCTT TCG	57
FOXQ1	GAGAAAAAGCCCAGC GGAAGA	AGCACCTTGACGAAG CAGTC	57
CEMIP	GCTCTTGAGTTGCATG GACA	ACCGCGTTCAAATACT GGAC	55
GAPDH	AATCCCATCACCATCT TCCA	TGAGTACGTCGTGGAG TCCA	53

Supplementary Table S2. Top 10 up-regulated genes in CRC (GSE21815)

T/N ratio	GENE_SYMBOL	GENE_NAME
108.65	*DPEP1*	dipeptidase 1 (renal)
67.72	*KRT80*	keratin 80
59.75	*FABP6*	fatty acid binding protein 6, ileal
56.90	*NKD2*	naked cuticle homolog 2 (Drosophila)
56.08	*FOXQ1*	forkhead box Q1
51.98	*CEMIP*	cell migration inducing hyaluronan binding protein (also called KIAA1199)
41.53	*ETV4*	ets variant 4
31.95	*TESC*	tescalcin
30.61	*FUT1*	fucosyltransferase 1 (galactoside 2-alpha-L-fucosyltransferase, H blood group)
30.49	*GAS2*	growth arrest-specific 2

Supplementary Table S3. Univariate and multivariate Cox regression analysis of the 10 CRC-associated genes

**Gene Name**	**Univariate analysis**			**Multivariate analysis**		
	Patient number (N)	Hazard ratio (95% CI)	P	Patient number (N)	Hazard ratio (95% CI)	P
DPEP1						
Low expression	83	0.430	**0.003**	83	0.430	0.003
High expression	143	(0.246-0.751)		143	(0.246-0.750)	
KRT80						
Low expression	211	4.082	**<0.001**	211	0.6067	0.490
High expression	15	(1.907-8.740)		15	(0.147-2.505)	
FABP6						
Low expression	147	0.6723	0.208			
High expression	79	(0.3624-1.247)				
NKD2						
Low expression	44	1.825	0.168			
High expression	182	(0.777-4.289)				
FOXQ1						
Low expression	201	1.727	0.158			
High expression	25	(0.809-3.685)				
CEMIP						
Low expression	152	0.611	0.128			
High expression	74	(0.324-1.152)				
ETV4						
Low expression	152	0.536	0.068			
High expression	74	(0.274-1.046)				
TESC						
Low expression	167	2.083	**0.011**	167	0.849	0.631
High expression	59	(1.182-3.671)		59	(0.434-1.660)	
FUT1						
Low expression	137	1.408	0.230			
High expression	89	(0.805-2.463)				
GAS2						
Low expression	20	0.509	0.099			
High expression	206	(0.228-1.135)				

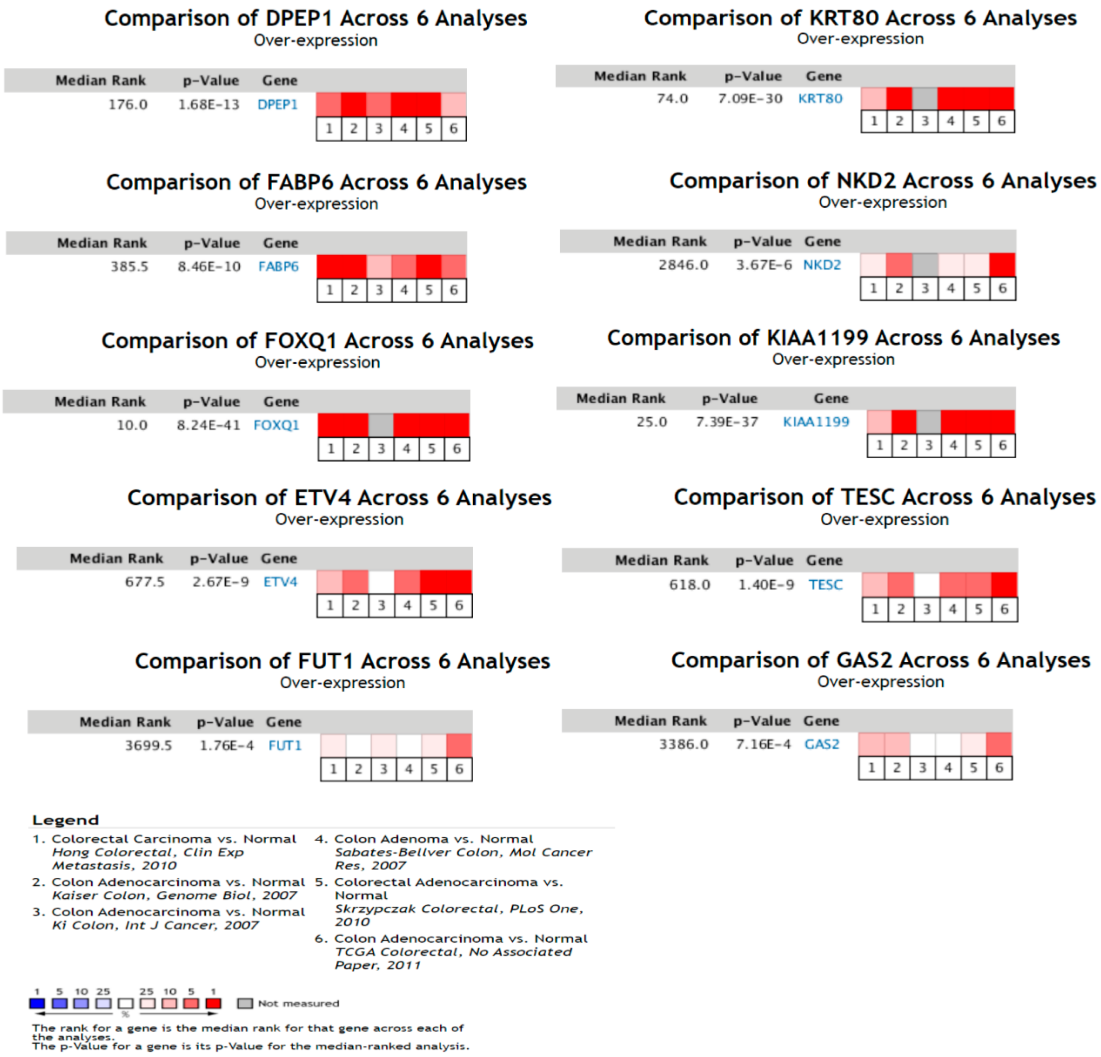

Figure S1. The expression status of top 10 upregulated CRC-associated genes in the total of 716 samples from six studies including, Hong, Kaiser, Ki, Sabates-Bellver, Skrzypczak, and TCGA databases in ONCOMINE by meta-analysis.

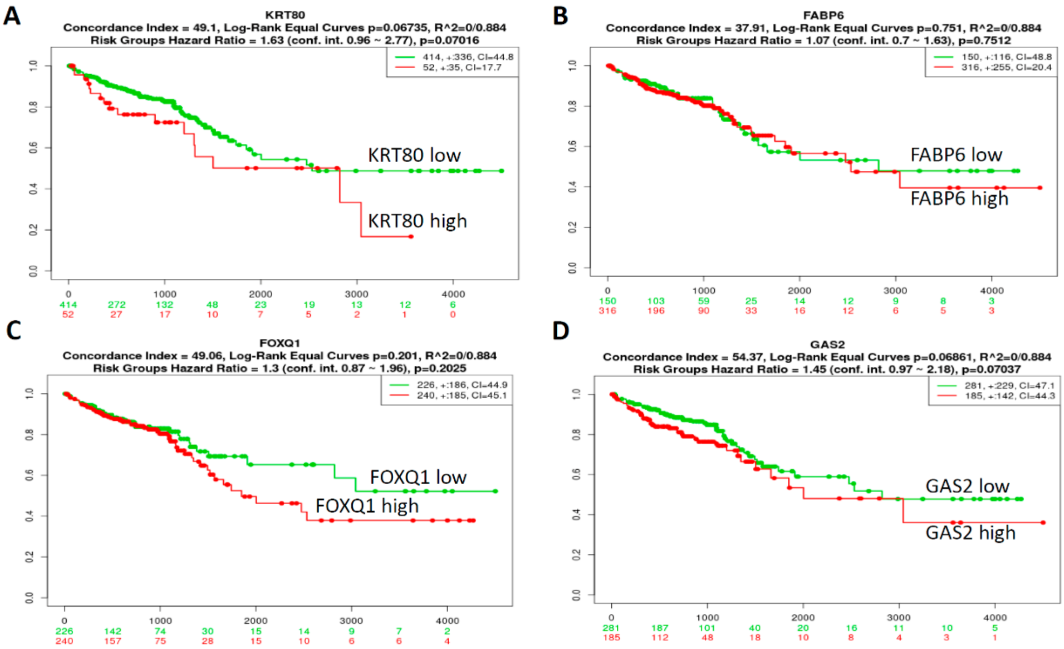

Figure S2. The prognostic value of mRNA expression level of 4 CRC-associated genes in patients with CRC. The relationship between KRT80 **(A)**, FABP6 **(B)**, FOXQ1 **(C)**, and GAS2 **(D)** expression and survival status in 466 TCGA-CRC patients.

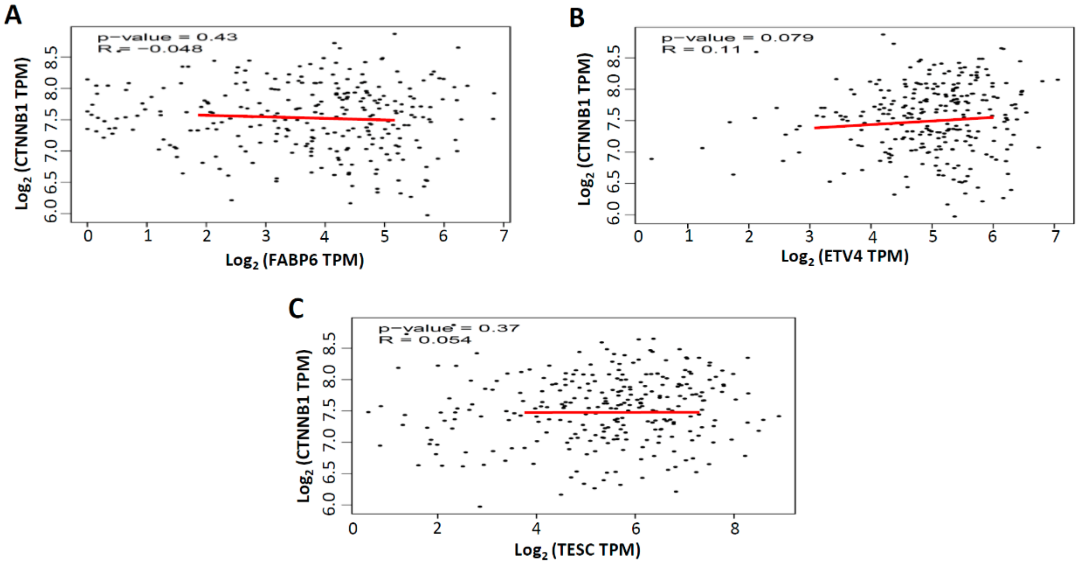

Figure S3. The correlation between CTNNB1 and 3 CRC-associated genes in CRC. **(A)-(C)** There was no correlation between CTNNB1 and FABP6, ETV4, or TESC in 275 CRC patients from the TCGA database.
